# 

**Published:** 1873-02

**Authors:** 


Vol. III.] FEBRUARY, 1873. [No. 2.

### THE SOUTHERN
### MEDICAL RECORD:

A MONTHLY JOURNAL OF

### PRACTICAL MEDICINE.

EDITED BY

T. S. POWELL, M.D.,

AND

W. T. GOLDSMITH, M.D.

*"QUICQUID PRÆCIPIES ESTO BREVIS."*

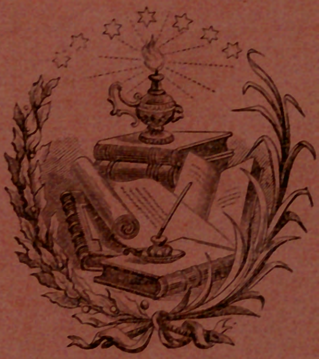

ATLANTA, GEORGIA:
Plantation Publishing Company's Press.
1873.

Terms, $2 50 Per Annum, in Advance. Single Number, 25 Cents.

